# Biomarkers in outpatient heart failure management; Are they correlated to and do they influence clinical judgment?

**DOI:** 10.1007/s12471-013-0503-y

**Published:** 2013-12-14

**Authors:** J. M. P. W. U. Peeters, S. Sanders-van Wijk, S. Bektas, C. Knackstedt, P. Rickenbacher, F. Nietlispach, R. Handschin, M. T. Maeder, S. F. Muzzarelli, M. E. Pfisterer, H. P. Brunner-La Rocca

**Affiliations:** 1 Department of Cardiology, Maastricht University Medical Center, CARIM, P. Debyelaan 25, PO box 5800, 6202 AZ Maastricht, the Netherlands; 2 University Hospital Bruderholz, Bruderholz, Switzerland; 3 University Hospital Basel, Basel, Switzerland; 4 University Hospital Berne, Berne, Switzerland; 5 Kantonsspital St Gallen, St Gallen, Switzerland; 6 Fondazione Cardiocentro Ticino, Lugano, Switzerland

**Keywords:** Heart failure, NT-proBNP, Natriuretic peptide, Biomarkers, Cystatin-C, Hs-CRP, Hs-TnT, GDF-15

## Abstract

**Aims:**

Heart failure (HF) management is complicated by difficulties in clinical assessment. Biomarkers may help guide HF management, but the correspondence between clinical evaluation and biomarker serum levels has hardly been studied. We investigated the correlation between biomarkers and clinical signs and symptoms, the influence of patient characteristics and comorbidities on New York Heart Association (NYHA) classification and the effect of using biomarkers on clinical evaluation.

**Methods and results:**

This post-hoc analysis comprised 622 patients (77 ± 8 years, 76 % NYHA class ≥3, 80 % LVEF ≤45 %) participating in TIME-CHF, randomising patients to either NT-proBNP-guided or symptom-guided therapy. Biomarker measurements and clinical evaluation were performed at baseline and after 1, 3, 6, 12 and 18 months. NT-proBNP, GDF-15, hs-TnT and to a lesser extent hs-CRP and cystatin-C were weakly correlated to NYHA, oedema, jugular vein distension and orthopnoea (ρ-range: 0.12–0.33; *p* < 0.01). NT-proBNP correlated more strongly to NYHA class in the NT-proBNP-guided group compared with the symptom-guided group. NYHA class was significantly influenced by age, body mass index, anaemia, and the presence of two or more comorbidities.

**Conclusion:**

In HF, biomarkers correlate only weakly with clinical signs and symptoms. NYHA classification is influenced by several comorbidities and patient characteristics. Clinical judgement seems to be influenced by a clinician’s awareness of NT-proBNP concentrations.

**Electronic supplementary material:**

The online version of this article (doi:10.1007/s12471-013-0503-y) contains supplementary material, which is available to authorized users.

## Introduction

Heart failure (HF) is a prominent health issue of the Western civilisation affecting up to 10 % of those aged over 75 years. Despite global consensus on treatment goals, HF management remains suboptimal, resulting in high mortality and hospitalisation rates [1]. In part, this may be due to difficulties in clinical evaluation, with symptoms and signs often being vague and undiscriminating [2]. In search for more objective clinical evaluation methods, B-type natriuretic peptide (BNP) and N-terminal pro-BNP (NT-proBNP) were suggested as biomarkers for assessment of the severity of HF. Measurements of BNP and NT-proBNP have proven to provide significant information regarding diagnostics and prognosis [3], reflecting endogenous responses to cardiac overload and neuro-hormonal stimulation [4]. Nonetheless, the sole measurement of natriuretic peptides may be insufficient, as they are not specific for cardiac overload but reflect multiple cardiac and non-cardiac pathologies [5] and are influenced by various other factors such as body mass index (BMI) [6], age [7], gender [8], and renal function [9]. On the other hand, HF pathophysiology is complex, involving various pathways. Thus, biomarkers other than natriuretic peptides such as high-sensitivity troponin T (hsTNT) reflecting myocyte injury [10], growth differentiation factor-15 (GDF15), associated with cardiac hypertrophy and dilated cardiomyopathy [11], serum cystatin C reflecting renal function [12] and high sensitivity C-reactive protein (hsCRP) indicative of inflammation [13], could be valuable in the evaluation of HF patients. Although implementation of biomarkers in the clinical care of HF has been under extensive investigation, correlations between biomarkers and signs and symptoms of HF, though important for further clinical implementation, remain largely unknown and limited to New York Heart Association (NYHA) class and natriuretic peptides [14–21]. Also, clinical assessment may not be completely independent of knowledge on biomarkers, as suggested for the intervention arm in studies investigating NT-proBNP-guided treatment [22] which are usually single blinded. Therefore, we investigated correlations between biomarkers and signs and symptoms of HF, assessed the effect of patient characteristics on symptoms and addressed the effect of knowing NT-proBNP levels on clinical judgement in the Trial of Intensified versus standard Medical therapy in Elderly patients with Congestive Heart Failure (TIME-CHF) [23].

## Methods

### Study design and population

Study design and methods of TIME-CHF have been described elsewhere [23]. In brief, TIME-CHF was a randomised, controlled, single-blinded, multicentre trial conducted in 15 clinical centres in Switzerland and Germany. Chronic HF patients older than 60 years, with dyspnoea defined by NYHA class ≥ 2 under therapy, a history of HF-related hospitalisation within the last year before inclusion, NT-proBNP levels of ≥ 400 ng/l in patients aged 60–74 years and ≥ 800 pg/ml in patients aged ≥ 75 years, BMI ≤ 35 kg/m^2^ and serum creatinine ≤ 220 μmol/l were included.

A total population of 622 patients, 499 with reduced (i.e. left-ventricular ejection fraction (LVEF) ≤45 %) and 123 with preserved LVEF of >45 %, was included between January 2003 and December 2006. Patients were randomised to either NT-proBNP-guided or symptom-guided management. Patients were followed up at pre-specified visits after 1, 3, 6, 12 and 18 months in the outpatient clinics of the participating centres. NT-proBNP levels were measured in all patients at every visit, although investigators were only provided with results in the NT-proBNP-managed patients. Investigators performed clinical assessment on all patients at every visit. This included NYHA classification for assessment of dyspnoea and severity of oedema, rales, central venous pressure (CVP) and orthopnoea, which were ranked between 0 and 3 (none, minor, moderate, severe).

Medical treatment was prescribed according to current guidelines by predefined escalation rules [23]. Treatment goals were defined as either a reduction of clinical symptoms to NYHA ≤ 2 or an additional reduction of NT-proBNP below the age-specific target values ≤ 400 ng/l for patients aged ≤ 75 years and ≤ 800 ng/l for patients aged ≥ 75 years for symptom-guided or NTpro-BNP-guided management respectively. Judgement regarding the possibility of treatment escalation was left to the investigator and based on clinical status and presence of significant side effects.

Biomarkers were measured by the use of commercially available assays by Roche diagnostics (Elecsys 2010).

### Statistical analysis

Baseline characteristics are presented as mean ± standard deviation SD, median (interquartile range (IQR)) or frequencies and percentages, as appropriate. Correlations between NYHA class, severity of other signs and symptoms and biomarkers were investigated using non-parametric Spearman’s correlation (ρ). Correlations were assessed in the entire population and separately for the two treatment groups to assess whether knowledge of NT-proBNP concentrations influenced clinical judgement. To further investigate this effect, we split patients into early- and late-included patients, i.e. included before or after 21 January 2005, respectively, to see whether study duration affected the influence of NT-proBNP concentrations on clinical judgement. Other subgroup analyses addressed the influence of age, systolic dysfunction, BMI, kidney disease, chronic obstructive pulmonary disease (COPD), stroke, peripheral artery disease, pulmonary artery hypertension, cancer and presence or absence of ≥2 comorbidities on the correlation between NT-proBNP and clinical signs and symptoms. Logistic regression was performed to identify characteristics or comorbidities that influence NYHA classification at baseline. Statistically significant variables were entered into multivariate logistic regression analysis. First, patient characteristics were entered into the model using a stepwise backward procedure using *p* < 0.05 for inclusion and *p* < 0.1 for exclusion in the model. Then, biomarkers were entered similarly in the model.

Differences between correlations in treatment groups and other subgroups were investigated using the formula described by Cohen and Cohen (1983) [24] for comparison of independent correlation coefficients, following Fischer’s r-to-z transformation. Other statistical calculations were performed with use of the SPSS statistical package version 20.0 (SPSS Inc, Chicago Illinois).

## Results

### Baseline characteristics

Baseline characteristics are depicted in Table **1**. This study comprised a highly symptomatic (i.e. 76 % NYHA ≥3) elderly HF population, with a high prevalence of comorbidities. The majority of patients suffered from systolic dysfunction. Of the patients, 70 either died or were lost to follow-up at month 1, 40 at month 3, 29 at month 6, 56 at month 12, and 26 at month 18. Thus, a total of 222 (36 %) did not complete the total study period of 18 months, of whom 82 withdrew consent and 140 died.

**Table 1:** Baseline characteristics

Age, mean (SD), y	76.9 ± 7.6
Male, n (%)	369 (59 %)
Body mass index, mean (SD)	25.6 ± 4.44
Systolic dysfunction, n (%)	499 (80.2 %)
Primary cause of heart failure, n (%)	
Coronary artery disease	330 (53.1 %)
Hypertensive heart disease	173 (27.8 %)
Dilated cardiomyopathy	89 (14.3 %)
Valvular heart disease	23 (3.7 %)
Other	7 (1.1 %)
Biomarkers	
N-terminal BNP, median (IQR), ng/l	3836 (1916–6905)
HsTNT, median (IQR), pg/ml	32.5 (18.6–59.8)
Cystatin-c, median (IQR), pg/ml	1.79 (1.44–2.20)
Creatinine, mean (SD) μmol/l	116 ± 38
HsCRP, median (IQR), pg/ml	7.7 (2.8–18.0)
Medical history, n (%)	
Diabetes	222 (35.7 %)
Hypertension	462 (74.3 %)
Dilated cardiomyopathy	94 (15.1 %)
Kidney disease	355 (57.1 %)
Comorbidity (≥2)	459 (73.8 %)
Atrial fibrillation	210 (33.8 %)
COPD	124 (19.9 %)
Stroke	52 (8.4 %)
Peripheral artery disease	124 (19.9 %)
Pulmonary artery hypertension	50 (8 %)
Anaemia	175 (28.1 %)
Cancer	86 (13.8 %)
Clinical examination, n (%)	
Orthopnoea (0–3)	197/225/144/53 (32/36/23/9 %)
Oedema (0–3)	357/103/93/64 (58/17/15/10 %)
Rales (0–3)	340/197/67/16 (55/32/11/2 %)
Jugular vein distention (0–3)	237/161/119/92 (39/26/20/15 %)
NYHA (II, III, IV)	149/388/85 (24/62/14 %)

### Correlations between NT-proBNP and clinical signs

Significant though weak correlations were identified between NT-proBNP and clinical signs and symptoms, at all visits. The strongest correlations were identified with NYHA class and jugular vein distention, respectively (Table 2). The correlation between NYHA class and NT-proBNP was largely independent of patient characteristics and comorbidities (data not shown).

**Table 2:** Spearman correlations (ρ) between biomarkers and clinical signs and symptoms (ρ)

	Baseline	Visit 1 month	Visit 3 months	Visit 6 months	Visit 12 months	Visit 18 months
NT-proBNP						
NYHA	0.22^**^	0.26^**^	0.28^**^	0.23^**^	0.32^**^	0.33^**^
Oedema	0.17^**^	0.18^**^	0.18^**^	0.18^**^	0.21^**^	0.20^**^
Rales	0.17^**^	0.16^**^	0.09	0.18^**^	0.17^**^	0.22^**^
JVD	0.23^**^	0.28^**^	0.25^**^	0.27^**^	0.29^**^	0.37^**^
Orthopnoea	0.13^**^	0.23^**^	0.23^**^	0.28^**^	0.24^**^	0.22^**^
Cystatin-C						
NYHA	0.21^**^	0.22^**^	0.25^**^	0.20^**^	0.20^**^	0.34^**^
Oedema	0.15^**^	0.21^**^	0.22^**^	0.17^**^	0.18^**^	0.23^**^
Rales	0.10^*^	0.09	0.10^*^	0.16^**^	0.10	0.16^**^
Jugular vein	0.12^**^	0.20^**^	0.23^**^	0.11^*^	0.22^**^	0.24^**^
Orthopnoea	0.15^**^	0.18^**^	0.20^**^	0.20^**^	0.23^**^	0.23^**^
hsTNT						
NYHA	0.22^**^	0.23^**^	0.25^**^	0.21^**^	0.27^**^	0.33^**^
Oedema	0.07	0.20^**^	0.18^**^	0.19^**^	0.22^**^	0.21^**^
Rales	0.17^**^	0.15^**^	0.12^**^	0.14^**^	0.19^**^	0.13^*^
JVD	0.19^**^	0.18^**^	0.24^**^	0.22^**^	0.28^**^	0.25^**^
Orthopnoea	0.18^**^	0.19^**^	0.19^**^	0.24^**^	0.29^**^	0.15^**^
hsCRP						
NYHA	0.14^**^	0.19^**^	0.16^**^	0.07	0.12^*^	0.12^*^
Oedema	0.18^**^	0.19^**^	0.12^*^	0.19^**^	0.15^**^	0.19^**^
Rales	0.19^**^	0.18^**^	0.08	0.02	0.03	0.17^**^
JVD	0.13^**^	0.15^**^	0.15^**^	0.10^*^	0.10^*^	0.02
Orthopnoea	0.13^**^	0.18^**^	0.13^**^	0.11^*^	0.22^**^	0.11^*^
GDF15						
NYHA	0.24^**^	0.22^**^	0.26^**^	0.23^**^	0.29^**^	0.31^**^
oedema	0.18^**^	0.22^**^	0.19^**^	0.16^**^	0.18^**^	0.15^**^
Rales	0.14^**^	0.12^**^	0.12^**^	0.16^**^	0.15^**^	0.16^**^
JVD	0.30^**^	0.28^**^	0.32^**^	0.19^**^	0.29^**^	0.32^**^
Orthopnoea	0.17^**^	0.17^**^	0.23^**^	0.22^**^	0.25^**^	0.24^**^

*p < 0.05, **P < 0.01. *JVD* jugular vein distention

### Correlations between other biomarkers and NYHA class

All biomarkers were weakly, though significantly, correlated with NYHA class at most visits, though less than with NT-proBNP. Correlations between NYHA and hsTNT, cystatin C and GDF 15, respectively, were comparable. Correlations between hsCRP and NYHA were the weakest and not significant at all visits (Table 2).

### Influence of NT-proBNP-guided therapy

**Fig. 1:**
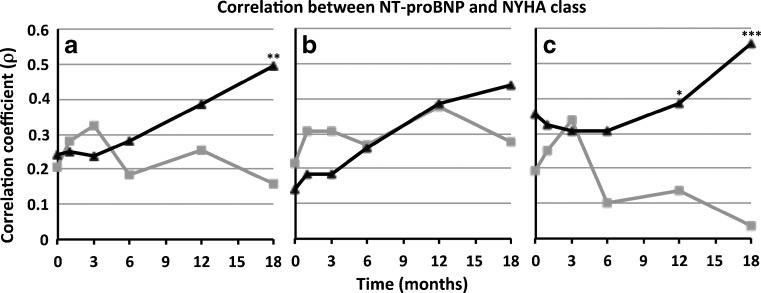
Spearman’s correlation (ρ) between NT-proBNP and NYHA divided by treatment group in all patients (**a**), early-included patients (**b**) and late-included patients (**c**); NT-proBNP guided (*black*), clinically guided (*grey*) * = *P* < 0.05; ** = *P* < 0.001, *** = *P* < 0.0001

Correlations between NT-proBNP and NYHA became stronger in the NT-proBNP-guided compared with the symptom-guided group as the study progressed (Fig. 1a). Interestingly, there was no significant influence of treatment group allocation in the early-included patients (Fig. 1b), whereas the influence of treatment group allocation on the correlation between NT-proBNP and NYHA was most pronounced in patients included in the second half of the study (Fig. 1c). Correlations between oedema and NT-proBNP were stronger in the NT-proBNP guided at the 18-month visit, whereas the correlation between JV distention and NT-proBNP was stronger in the clinically guided at the 3-month visit (Supplemental Fig. 1 A-D). Correlations between other signs and symptoms did not differ between the treatment groups. Correlations between other biomarkers and NYHA classification overall did not differ between treatment groups, besides GDF15 and NYHA, which correlated stronger in the NT-proBNP-guided group at the 3-month visit (Supplemental Fig. 2 A-D).

### Predictors of NYHA class

Several patient characteristics such as BMI, gender and several comorbidities were found to be related to NYHA class ≥3 (Table 3). Multivariable analysis indicated that age, female gender, BMI, anaemia and pulmonary artery hypertension (PAH) were associated with NYHA class ≥3 (Table 3). The stepwise addition of biomarkers to this model showed that only NT-proBNP was an independent predictor of NYHA class ≥ 3. GDF15 was of borderline significance. After NT-proBNP was added to the model, age and PAH were no longer independent predictors of NYHA class ≥ 3 (Table 3).

**Table 3:** Univariate and multivariate logistic regression on the association of patient characteristics, comorbidities and biomarkers, with a low functional class NYHA ≥3

	Univariate			Patient characteristics			Characteristics and biomarkers		
	OR	95 % C.I	*P*	OR	95 % C.I	*P*	OR	95 % C.I	*P*
Biomarkers									
BNP-lg10	3.70	2.21 – 6.18	<0.0001				3.15	1.59 – 6.24	0.001
Cystatin-C	2.35	1.56 – 3.55	<0.0001						
hsCRP-lg10	1.58	1.14 – 2.20	0.006						
hsTnT-lg10	2.27	1.14 – 3.66	0.001						
GDF15-lg10	7.49	3.14 – 17.83	<0.0001				2.84	0.95 – 8.46	0.061
Characteristics									
Female	2.05	1.37 – 3.06	<0.0001	1.86	1.21 – 2.85	0.005	1.83	1.11 – 3.00	0.017
Age	1.05	1.02 – 1.08	<0.0001	1.04	1.02 – 1.07	0.002	1.03	1.00 – 1.06	0.098
BMI			0.058			0.009			0.018
BMI <20	0.35	0.16 – 0.77	0.009	0.24	0.10 – 0.54	0.001	0.21	0.08 – 0.55	0.002
BMI 20–25	0.63	0.34 – 1.16	0.138	0.54	0.28 – 1.02	0.056	0.52	0.26 – 1.06	0.071
BMI 25–30	0.54	0.29 – 0.99	0.048	0.53	0.28 – 1.01	0.053	0.60	0.29 – 1.21	0.153
Syst. dysfunction	0.60	0.39 – 1.00	0.048						
Comorbidities									
COPD	1.27	0.93 – 1.73	0.131						
Stroke	0.94	0.49 – 1.81	0.854						
Kidney disease	1.43	0.99 – 2.07	0.057						
PAHT	3.03	1.18 – 7.78	0.021	2.73	1.04 – 7.15	0.041	2.70	0.91 – 8.00	0.074
Anaemia	2.06	1.30 – 3.26	0.002	1.98	1.23 – 3.19	0.005	1.53	0.88 – 2.65	0.129
Cancer	1.13	0.65 – 1.95	0.663						
>2 comorbidities	1.54	1.03 – 2.31	0.034						
PAOD	0.88	0.56 – 1.39	0.589						

*BMI* body mass index; *COPD* chronic obstructive pulmonary disease; *PAHT* pulmonary artery hypertension, *PAOD* peripheral artery occlusive disease

## Discussion

This study demonstrates that both new and established biomarkers are correlated only weakly with clinical signs and symptoms of HF, that clinical signs and symptoms are influenced by several comorbidities and patient characteristics independent of HF and that the use of biomarkers may influence clinical judgement.

HF has a highly complex, multi-organ pathophysiology. Correlations between clinical signs as well as symptoms and biomarkers reflecting these pathophysiological mechanisms were found to be weak. This may have various reasons. For instance, serum levels of biomarkers as NT-proBNP respond rapidly to changes in disease severity [25] and have short half-times [26], whereas clinical signs and symptoms may be less sensitive due to systemic compensatory reactions [27]. Also, patient characteristics and diseases other than HF may significantly influence the severity of symptoms. Indeed, NYHA classification was found to be influenced by comorbidities and patient characteristics such as anaemia, PAH, presence of multiple comorbidities as well as age, BMI and gender. Still, some patient characteristics and comorbidities may influence biomarkers and symptoms likewise and not independently of each other. Also COPD, which logically should have a negative effect on NYHA classification, was not identified as a significant predictor of low functionality in our study. These findings highlight the complexity of the clinical assessment and the fact that none of the signs and symptoms are really specific for HF.

Finally, clinical assessment is subjective and therefore has important limitations. NYHA classification has large inter-observer variability, resulting in the finding that classification of patients in class II or III may depend on little more than chance [28]. Other clinical symptoms have hardly been studied with respect to their accuracy and reproducibility. However, previous studies in acute decompensated HF showed their limitation for correct clinical assessment and more objective tests including measurements of BNP and NT-proBNP may be more accurate [29]. Our findings are in agreement with these results, as we showed that biomarkers do not merely reflect signs or symptoms of HF and may thus provide additional information. This suggests that biomarkers may add to clinical judgement in HF patients in clinically stable condition.

A number of studies, in agreement with our results, have reported significant associations between natriuretic peptides and NYHA classification [14–21]. However, only two studies were identified that reported the specific correlations between NT-proBNP and NYHA classification. Those reported stronger correlations than we identified [18, 19]. This disagreement may have several reasons. Population sizes were significantly smaller than in our study [18, 19]. Also, population characteristics were different, the patients being younger and having lower NYHA classification scores [18, 19]. We found that correlations between NYHA class and NT-proBNP were weaker with increasing age. None of these studies reported extensively on comorbidities [18, 19] and one did not report BMI index [19], which may have influenced NYHA classification. In the latter study, patients also did not have an objective proof of HF [19], nor was it reported whether NT-proBNP measurements were blinded for investigators performing NYHA classification [19], which may have influenced their clinical judgement as suggested by our results.

As the study progressed, correlations between NT-proBNP and NYHA class became significantly stronger in the NT-proBNP-guided, but not the symptom-guided group, where investigators were blinded to the NT-proBNP results. This strongly suggests that clinical judgement may be influenced by knowledge of NT-proBNP results. Interestingly, the difference in correlation is even more pronounced in late-included patients, while in early-included patients this relationship is virtually absent. Therefore, it seems that clinicians become more influenced in their clinical judgement by knowledge of biomarker concentrations, as they become more familiar with their interpretation. The correlation between other biomarkers and NYHA class were not influenced by study duration. These were not used in clinical management and investigators were blinded to them in both treatment groups. The relationship was also far less with other signs and symptoms of HF. This might be explained, as assessment of oedema and especially rales, jugular vein distention and orthopnoea may be less vulnerable to subjective interpretation of clinicians. However, this assumption was not properly tested and should be investigated prospectively.

As this study is a post-hoc analysis, it inevitably has limitations and should be valued as exploratory. Also investigators participating in the trial may be more accustomed to using biomarkers in their clinical practice. However, if this assumption were true, it would further reduce the already weak correlation found.

In conclusion, biomarkers including NT-proBNP were only weakly correlated to signs and symptoms. At first glance, this might seem controversial and could raise questions regarding the importance of biomarkers in HF management. However, our findings may actually support the utility of biomarkers in the clinical setting, as they may provide additional information rather than merely reflecting signs and symptoms and confirm what is already known. Importantly, our data suggest that knowledge of biomarkers may significantly influence clinical judgement, which may have important implications regarding the use of biomarkers in clinical practice.

## Funding

This study was sponsored by the Horten Research Foundation (Lugano, Switzerland; >55 % of the study’s budget), as well as by smaller unrestricted grants from AstraZeneca Pharma, Novartis Pharma, Menarini Pharma, Pfizer Pharma, Servier, Roche Diagnostics, Roche Pharma, and Merck Pharma. The sponsors had no role in the design and conduct of the study; collection, management, analysis, and interpretation of the data; or in the preparation of the manuscript.

## Electronic supplementary material

Below is the link to the electronic supplementary material.

Supplemental Figure 1 Correlation (ρ) between NT-proBNP and clinical signs and symptoms, NT-proBNP guided (black), clinically guided (grey). From left to right: total patients, early-included patients and late-included patients* = *P* < 0.05. (JPEG 52.6 KB)

High resolution image (TIFF 10.7 MB)

Supplemental Figure 2 Correlation (ρ) between biomarkers and NYHA classification, NT-proBNP guided (black), clinically guided (grey). From left to right: total patients, early-included patients and late-included patients* = *P* < 0.05. (JPEG 52.4 KB)

High resolution image (TIFF 10.6 MB)
