# Supplementary figures and images for: Biomarkers in outpatient heart failure management; Are they correlated to and do they influence clinical judgment?
Source: Neth Heart J. 2013 Dec 14;22(3):115–21. doi: 10.1007/s12471-013-0503-y (PMC3931853; doi:10.1007/s12471-013-0503-y)

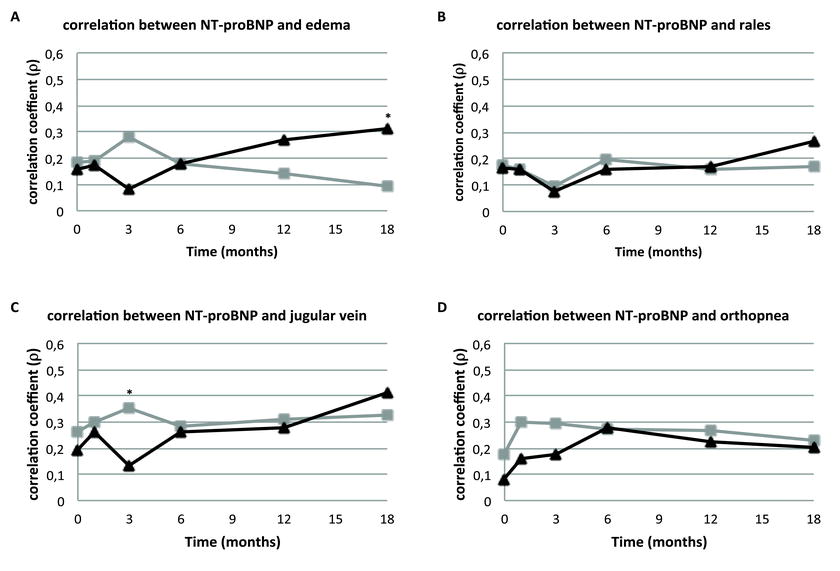

Supplement: Supplementary file 1 — Correlation (ρ) between NT-proBNP and clinical signs and symptoms, NT-proBNP guided (black), clinically guided (grey). From left to right: total patients, early-included patients and late-included patients* = P < 0.05. (JPEG 52.6 KB) [file 12471_2013_503_Fig1_ESM.jpg]

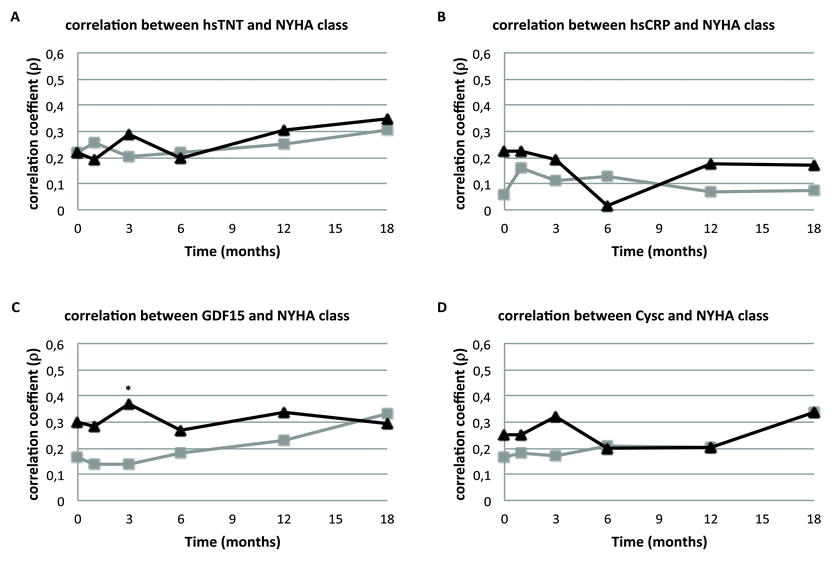

Supplement: Supplementary file 3 — Correlation (ρ) between biomarkers and NYHA classification, NT-proBNP guided (black), clinically guided (grey). From left to right: total patients, early-included patients and late-included patients* = P < 0.05. (JPEG 52.4 KB) [file 12471_2013_503_Fig2_ESM.jpg]
